# Targeting Pancreatic Cancer Cells with Peptide-Functionalized Polymeric Magnetic Nanoparticles

**DOI:** 10.3390/ijms20122988

**Published:** 2019-06-19

**Authors:** Xiuliang Zhu, Nan Lu, Ying Zhou, Shaoyan Xuan, Jiaojiao Zhang, Francesca Giampieri, Yongping Zhang, Fangfang Yang, Risheng Yu, Maurizio Battino, Zuhua Wang

**Affiliations:** 1 Zhejiang University School of Medicine, Hangzhou 310009, China; zhuxiul@zju.edu.cn (X.Z.); 2317075@zju.edu.cn (N.L.); 2 College of Pharmaceutical Sciences, Guizhou University of Traditional Chinese Medicine, Guiyang 550025, China; yingzhou71@126.com (Y.Z.); gzzhyp@126.com (Y.Z.); yff19771128@126.com (F.Y.); 3 College of Pharmaceutical Sciences, Zhejiang University, Hang Zhou 310058, China; xsy24506@163.com; 4 Department of Clinical Sciences, Faculty of Medicine, Università Politecnica delle Marche, 60131 Ancona, Italy; zh.jojo@yahoo.com (J.Z.); f.giampieri@univpm.it (F.G.); 5 Nutrition and Food Science Group, Department of Analytical and Food Chemistry, CITACA, CACTI, University of Vigo—Vigo Campus, 32004 Ourense, Spain

**Keywords:** pancreatic cancer, CKAAKN peptide, MRI contrast agent, polymer

## Abstract

Pancreatic cancer is a concealed and highly malignant tumor, and its early diagnosis plays an increasingly weighty role during the course of cancer treatment. In this study, we developed a polymeric magnetic resonance imaging (MRI) nanoplatform for MRI contrast agents. To improve tumor-targeting delivery of MRI contrast agents, we employed a pancreatic cancer targeting CKAAKN peptide to prepare a peptide-functionalized amphiphilic hyaluronic acid–vitamin E succinate polymer (CKAAKN–HA–VES) for delivering ultra-small superparamagnetic iron oxide (USPIO), namely, CKAAKN–HA–VES@USPIO. With the modification of the CKAAKN peptide, CKAAKN–HA–VES@USPIO could specifically internalize into CKAAKN-positive BxPC-3 cells. The CKAAKN–HA–VES@USPIO nanoparticles presented a more specific accumulation into pancreatic cancer cells than normal pancreatic cells, and an obvious decrease in signal intensity was observed in CKAAKN-positive BxPC-3 cells, compared with CKAAKN-negative HPDE6-C7 cells and non-targeting HA–VES@USPIO nanoparticles. The results demonstrated that our polymeric MRI nanoplatform could selectively internalize into CKAAKN-positive pancreatic cancer cells by the specific binding of CKAAKN peptide with pancreatic cancer cell membrane receptors, which provided a novel polymeric MRI contrast agent with high specificity for pancreatic cancer diagnosis, and makes it a very promising candidate for magnetic resonance imaging contrast enhancement.

## 1. Introduction

Pancreatic cancer is concealed and highly malignant, with a dismal prognosis and few effective therapies [1,2]. As a malignant tumor, pancreatic cancer remains a major unsolved health problem and is the leading cause of cancer deaths in China due to the late diagnosis and limited response to treatment, although it accounts for approximately 2 to 3 percent of all cancers [3,4]. In recent years, the incidence of pancreatic cancer has increased worldwide, and almost all patients who have been afflicted with pancreatic cancer have suffered metastases and death [5]. Therefore, the early diagnosis of pancreatic cancer plays an increasingly substantial role during the course of cancer treatment, which enables a quick response to the development of the cancer and rapid development of a reasonable therapeutic strategy for patients [6]. Currently, pancreatic cancer is mainly diagnosed through imaging and the detection of tumor markers in serum. The carbohydrate antigen CA19-9 is currently the most widely used and recognized tumor marker in the diagnosis of pancreatic cancer [7,8], but it can also be detected in benign diseases, such as chronic pancreatitis, because of the lack of sufficient sensitivity and specificity. Various imaging modalities such as computed tomography (CT) [9], magnetic resonance imaging (MRI) [10], endoscopic ultrasound (EUS) [11], and positron emission computed tomography (PET) [12] have become increasingly important and are commonly used to diagnose pancreatic cancer, but it is difficult for these imaging methods to accurately diagnose pancreatic tumors less than 2 cm in diameter. Unfortunately, most patients in early cancer stages are not diagnosed in a timely manner, which prevents patients from the opportunity for receiving the best treatment. Therefore, it is vitally important to realize accurate early diagnosis and treatment of pancreatic cancer. Recently, targeted molecular imaging techniques have developed as promising cancer imaging strategies in modern diagnostics because they provide accurate and specific information regarding the diagnosis of diseases [13], which make targeted molecules excellent contrast agents for molecular imaging purposes.

In recent years, polymer micelles have become a novel carrier for drug and imaging contrast agent delivery [14,15]. Polymer micelles can self-assemble in aqueous solution to form a distinct core–shell structure with hydrophobic cores and hydrophilic shells, and the enhanced penetration and retention effect allows the polymeric carrier system to passively target the tumor site and increase the concentration of an imaging contrast agent at the tumor site.

Along with the trend of accurate diagnosis and treatment of cancer, to improve the diagnostic accuracy of early pancreatic cancer, it is crucial to develop targeted molecular probes with high sensitivity, high specificity, low toxicity, and minimal side effects. Hyaluronic acid (HA) is a natural glycosaminoglycan that is widely distributed in the human connective tissue matrix, animal skin, the vitreous body, cartilage tissue, and joint synovial fluid with good physicochemical properties, such as biodegradability and low immunogenicity. Hyaluronic acid molecules are rich in modifiable groups such as hydroxyl and carboxyl groups; therefore, various chemical reactions and functional modifications are possible, and these modifications are often used for carrier materials for the delivery of drugs and biocontrast agents [16].

CD44 is the principal cell surface receptor for HA, and it is widely present in many tumor cells such as pancreatic cancer cells, which suggests that it is promising and significant to use hyaluronan–CD44 interactions as potential targets for early cancer diagnosis [17,18]. Vitamin E succinate (VES) is one of the derivatives of vitamin E and has good stability. Currently, VES is widely used in the fields of medicine, food, and cosmetics as a vitamin E substitute. Numerous studies have shown that VES can effectively inhibit the growth of a variety of tumor cells in vitro and in vivo without toxicity and the inhibition of normal cell growth [19,20]. In this study, cystamine was used as a linker to link hydrophilic HA and lipophilic VES in the presence of carbodiimide and N-hydroxysuccinimide, and HA–VES polymer micelles were obtained. Experimental studies regarding the use of HA–VES polymer micelles as a molecular probe carrier have been rarely reported both locally and worldwide. Due to the high expression of the HA receptor on the surface of pancreatic cancer cells, we proposed HA–VES polymer micelles with a targeted recognition function to pancreatic cancer cells as an ideal molecular probe targeting vector. However, since HA receptors can also be abnormally expressed on the surface of other human tumor cells, their targeting ability is not sufficiently specific.

A tumor-homing peptide is an amino acid polypeptide with high affinity and specific targeting to a ligand screened by phage display technology, and it has the advantages of low molecular weight, easy synthesis, low immunogenicity, in vivo degradation products, abundant source material, and other characteristics [21,22]. In this study, the HA–VES polymer was used to load ultra-small superparamagnetic iron oxide (USPIO). To enhance tumor targeting, HA–VES@USPIO was further modified by a CKAAKN polypeptide, a novel phage-display tumor-homing peptide. The CKAAKN polypeptide is a linear amino acid that targets tumor cells and neovascular endothelial cells [23]. The mechanism of CKAAKN binding to tumor cells mainly simulates the specific binding between the Wnt protein and the pancreatic cancer cell membrane receptor through the Wnt signaling pathway and has almost no specific binding capacity to normal pancreatic tissue [24]. Therefore, the CKAAKN polypeptide is an ideal and promising target for tumor-targeted delivery in our system, and the specific binding and targeted magnetic resonance imaging of the synthesized polymeric magnetic nanoparticles with pancreatic cancer cells were further investigated in this study.

## 2. Results

### 2.1. Synthesis and Characteristics of the CKAAKN–HA–VES Polymer

**Figure 1:**
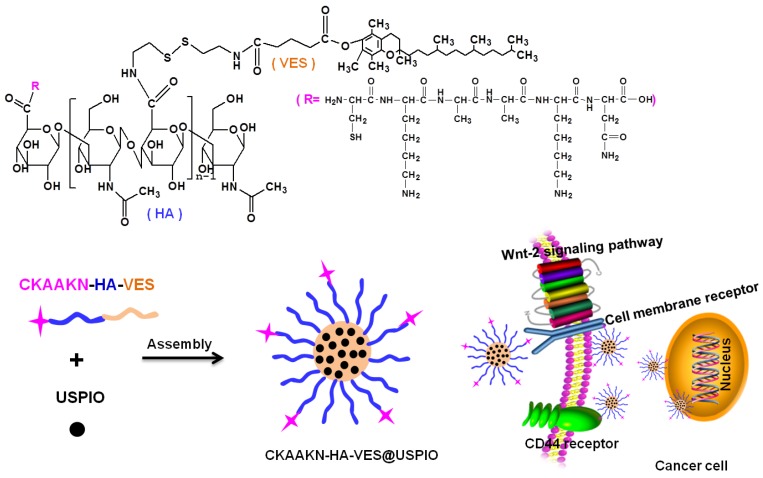
Preparation procedure of CKAAKN–HA–VES@USPIO and schematic illustration of BXPC-3 cells in vitro tracking.

**Figure 2:**
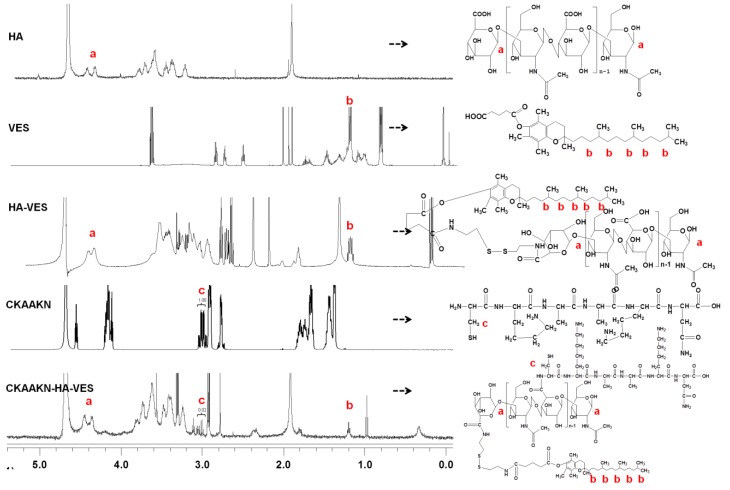
^1^H NMR spectra of HA, VES, HA–VES, CKAAKN, and CKAAKN–HA–VES.

The synthesis scheme of CKAAKN–HA–VES@USPIO and schematic illustration of BXPC-3 cells in vitro tracking is shown in Figure 1. The synthesis of CKAAKN–HA–VES was confirmed from the ^1^H NMR spectrum, Figure 2. The ^1^H NMR spectra of HA, VES, HA–VES, CKAAKN and CKAAKN–HA–VES were collected, and they are presented in this order from top to bottom. HA has a double peak at approximately 4.69–4.61 ppm, which was attributed to the methane hydrogen proton peak of the furan nucleus in HA. VES had a single peak at approximately 1.20–1.28 ppm, which was attributed to the methyne hydrogen proton peak of the alkane in VES. CKAAKN had a fingerprint characteristic peak at approximately 3.0–3.06 ppm, which was attributed to the methyne hydrogen proton peak of the amino thiopropionic acid in CKAAKN. The relative ratios of peak “c” integration values in CKAAKN and CKAAKN–HA–VES were 1.00 and 0.02, respectively (Figure 2), which indicated that the modification rate of the peptide was about 1.5%, that is, about 1.5 of every 100 carboxyl groups in the chain of HA polymer were replaced by CKAAKN peptide (on average). All three characteristic peaks appeared in the ^1^H NMR spectrum of CKAAKN–HA–VES, which indicates that the CKAAKN–HA–VES polymer was successfully synthesized, Figure 2.

**Figure 3:**
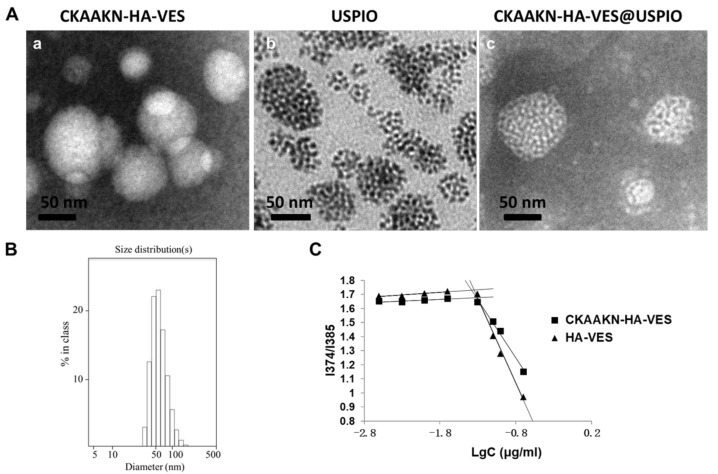
(**A**) TEM images of CKAAKN–HA–VES micelles, USPIO, and CKAAKN–HA–VES @USPIO nanoparticles (100,000×, bar = 50 nm); (**B**) size distribution of CKAAKN–HA–VES @USPIO; (**C**) variation of intensity ratio (I_1_/I_3_) versus concentration of CKAAKN–HA–VES (square) and HA–VES (triangle).

The aggregation behavior of CKAAKN–HA–VES and HA–VES nanoparticles in aqueous media was measured by fluorometry using pyrene as a fluorescent probe. The I_1_/I_3_ ratio, i.e., the intensity ratio between the first and third highest energy bands in the pyrene emission spectra (I_1_, em at 374 nm; I_3_, em at 385 nm), was used to determine the critical micelle concentration (CMC) of the polymer. The CMC value of the polymer was analyzed by Excel data processing software. The CMC values of CKAAKN–HA–VES and HA–VES nanoparticles in deionized water were approximately 48.08 and 44.51 μg/mL, respectively (Figure 3C). This result suggested that the effect of the CKAAKN modification on the CMC was generally negligible and that CKAAKN–HA–VES nanoparticles still had an excellent dispersity and ability to self-assemble in aqueous environments.

For the modification of the nanoparticles using the targeting CKAAKN peptide, the average diameter of the CKAAKN–HA–VES micelles was 87.5 ± 3.5 nm (Table 1), which was nearly consistent with the TEM results (Figure 3Aa), and there was no significant size difference compared with the HA–VES micelles (nm, Table 1). The TEM photographs demonstrate the successful entrapment of USPIO (Figure 3Ab) into CKAAKN–HA–VES.

### 2.2. Preparation and Characteristics of the CKAAKN–HA–VES@USPIO Nanoparticles

The synthesized CKAAKN–HA–VES@USPIO magnetic nanoparticles presented a specific core–shell structure where USPIO nanoparticles were encapsulated inside the polymer and served as a core and the CKAAKN–HA–VES polymer served as the shell, which was confirmed by TEM imaging (Figure 3Ac).

Due to the loading of USPIO nanoparticles, the mean diameter of CKAAKN–HA–VES@USPIO nanoparticles increased significantly from 87.5 ± 3.5 nm (CKAAKN–HA–VES, blank micelles) to 98.2 ± 4.3 nm, as determined using dynamic light scattering (Figure 3B and Table 1).

### 2.3. In Vitro Cellular Competitive Uptake Studies

**Figure 4:**
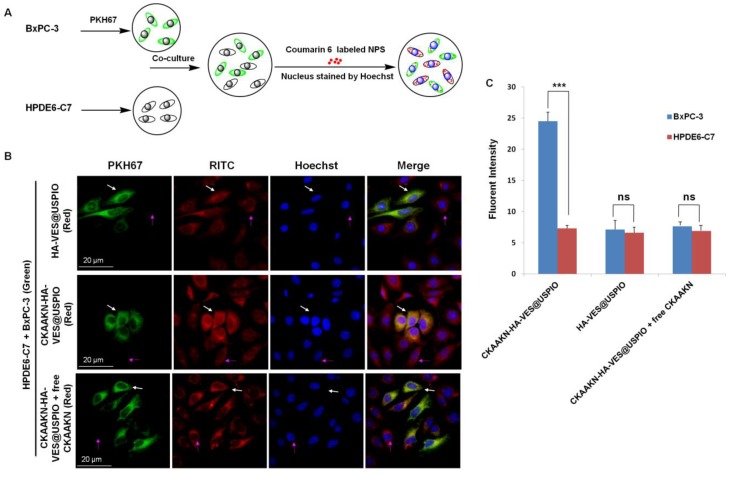
Cellular competitive uptake studies. (**A**) Schematic diagram of cellular competitive uptake of CKAAKN–HA–VES@USPIO and HA–VES@USPIO. (**B**) Confocal microscopy images of Coumarin 6-labeled CKAAKN–HA–VES@USPIO, HA–VES@USPIO, and CKAAKN–HA–VES@USPIO plus free CKAAKN after 1 h of incubation. BxPC-3 cells (the cytoplasmic membrane labeled with PKH67 fluorescent linker, green) cocultured with HPDE6-C7 cells were incubated with CKAAKN–HA–VES@USPIO, HA–VES@USPIO, and CKAAKN–HA–VES@USPIO plus free CKAAKN (red). The nuclei were all stained with Hoechst 33342. The white arrow stands for BxPC-3 cells; the pink arrow stands for HPDE6-C7cells. (**C**) The quantitative analysis based on the imaging in (**B**) by ImageJ software. *** corresponds to P < 0.001

To evaluate the specific cellular targeting of CKAAKN–HA–VES@USPIO, cocultured systems containing CKAAKN-positive and CKAAKN-negative cells were generated by incubating BxPC-3 and HPDE6-C7 cells in the same wells followed by further incubation with our pancreatic cancer-targeting magnetic nanoparticles (Figure 4A). As shown in Figure 4B, a significant difference in the cellular internalization of CKAAKN–HA–VES@USPIO nanoparticles was observed in the cocultured system containing BxPC-3/HPDE6-C7 cells. The increased cellular uptake of CKAAKN–HA–VES@USPIO nanoparticles in BxPC-3 cells (CKAAKN-positive, white arrows) was observed compared with that of HPDE6-C7 cells (CKAAKN-negative, pink arrows) during a short incubation time (i.e., 1 h). However, when the cocultured BxPC-3/HPDE6-C7 cells were incubated with HA–VES@USPIO nanoparticles (no CKAAKN conjugation), the results clearly showed that the HA–VES@USPIO nanoparticle uptake in both kinds of cells was not evident (Figure 4B).

CKAAKN-positive BxPC-3 cells exhibited increased CKAAKN–HA–VES@USPIO nanoparticle internalization compared with CKAAKN-negative HPDE6-C7 cells. The uptake of HA–VES@USPIO nanoparticles exhibited no significant difference between CKAAKN-positive and CKAAKN-negative cells.

Under the same conditions, further semiquantitative analysis of the competitive cellular uptake of the CKAAKN–HA–VES@USPIO nanoparticles in the BxPC-3/HPDE6-C7 cell cocultured system was performed by ImageJ software (Figure 4C). The relative fluorescence intensity, which indicates the amount of CKAAKN–HA–VES@USPIO cellular internalization, in BxPC-3 and HPDE6-C7 cells was 24.531 and 7.319 (*p* < 0.001) and became 7.661 and 6.921 after the addition of free CKAAKN peptide, respectively, indicating the increased cellular internalization of the CKAAKN–HA–VES@USPIO nanoparticles into CKAAKN-positive cells compared with CKAAKN-negative cells. After HA–VES@USPIO nanoparticles were added to the coculture system, the fluorescence values in BxPC-3 and HPDE6-C7 cells were 7.143 and 6.627, respectively. The result showed that adding excessive free CKAAKN peptide could block the binding of WNT receptor-positive BxPC-3 cells and resulted in the reduction of CKAAKN–HA–VES@USPIO uptake into the cells.

These results were consistent with those observed by confocal laser scanning microscopy (Figure 4B). Our data demonstrated that CKAAKN–HA–VES@USPIO nanoparticles could be specifically internalized by CKAAKN-positive cells via Wnt protein-mediated endocytosis.

### 2.4. In Vitro Cytotoxicity Studies

**Figure 5:**
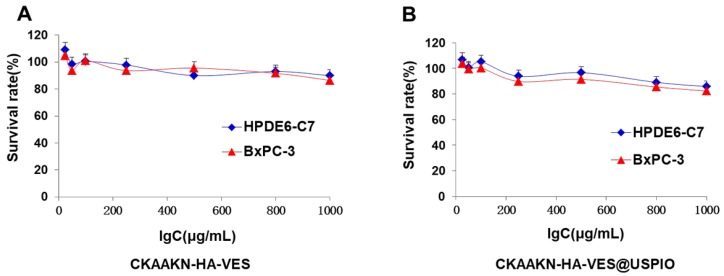
In vitro biosafety studies. BXPC-3 and HPDE6-C7 cells were incubated with blank CKAAKN–HA–VES polymer (**A**) and CKAAKN–HA–VES@USPIO (**B**) for 48 h, respectively. Cell viability was calculated by the percentage of living cells. Data represent the mean ± standard deviation (*n* = 3).

The in vitro biosafety of CKAAKN–HA–VES and CKAAKN–HA–VES@USPIO nanoparticles against BxPC-3 and HPDE6-C7 cells was further investigated by an MTT assay. As shown in Figure 5, blank CKAAKN–HA–VES polymer micelles and CKAAKN–HA–VES@USPIO nanoparticles showed nearly no cytotoxicity to BxPC-3 and HPDE6-C7 cells throughout the testing period. The viability of both BxPC-3 and HPDE6-C7 cells was over 80% for all concentrations of CKAAKN–HA–VES and CKAAKN–HA–VES@USPIO nanoparticles after 48 h of incubation.

### 2.5. In Vitro MR Imaging of CKAAKN–HA–VES@USPIO Nanoparticles

**Figure 6:**
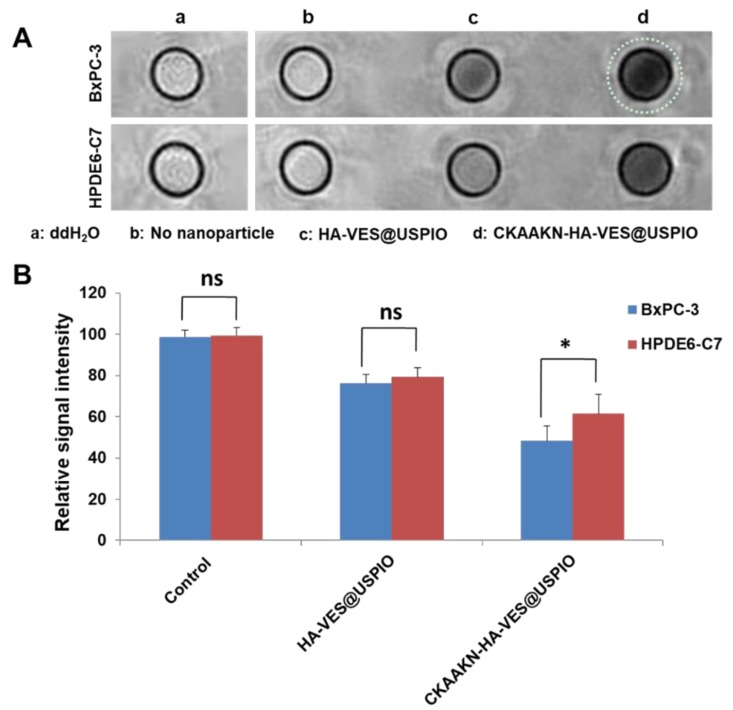
In vitro magnetic resonance (MR) imaging studies. (**A**) BxPC-3 cells or HPDE6-C7 cells were incubated with two different kinds of magnetic nanoparticles for 1 h, and then scanned with MR. (**B**) Signal intensity values of BxPC-3 cells or HPDE6-C7 cells in different groups were measured (* *p* < 0.05). Data represent the mean ± standard deviation (*n* = 3).

T2-weighted images of CKAAKN–HA–VES@USPIO and HA–VES@USPIO nanoparticles that were incubated with BxPC-3 and HPDE6-C7 cells for 1 h and their signal intensities are shown in Figure 6A. The most obvious decrease in the signal intensity can be seen in the CKAAKN–HA–VES@USPIO nanoparticles incubated with BxPC-3 cells, and the CKAAKN–HA–VES@USPIO nanoparticle signal intensity was significantly different from that of the other groups (*p* < 0.05) (Figure 6B). This may be attributed to the specific targeting ability of CKAAKN–HA–VES@USPIO nanoparticles to BxPC-3 cells.

## 3. Discussion

Polymeric micelles are promising nanocarrier systems for MRI contrast agent delivery, which might be an effective method to improve the diagnostic accuracy of early-stage cancer and reduce the systemic toxicity caused by conventional contrast agents. The detection of tumor markers plays an increasingly important role in early cancer diagnosis and may have important applications in early detection strategies [25,26]. However, tumor markers are not sufficiently specific, and only low rates of positive cases have been observed [27]. In recent years, MRI contrast agent-based cancer imaging and diagnosis have been developing, but its tumor-targeting, diagnostic accuracy, and biosafety are not promising. To overcome this problem, we used an amphiphilic HA–VES polymer, with a hydrophobicity/hydrophilicity ratio of 42.6%, which could self-assemble into polymeric micelles in water and display excellent internalization into cells, to encapsulate the MRI contrast agent USPIO (HA–VES@USPIO).

To improve the tumor-targeting delivery of USPIO, a pancreatic cancer-targeted CKAAKN peptide (a specific Wnt-2 mimetic), with specific binding to pancreatic cancer cell membrane receptors, was conjugated on the surface of the HA–VES@USPIO nanoparticles to increase tumor-selective accumulation. To obtain more effective diagnostic accuracy of pancreatic cancer, it is crucial that MRI contrast agents can be efficiently delivered into the targets. In this study, we developed a peptide-functionalized polymeric magnetic nanoparticle with a distinct core–shell structure, excellent biodegradability, tumor-targeting ability, and biosafety (Figure 2, Figure 3, Figure 4 and Figure 5).

For the safety and high efficiency of pancreatic cancer diagnosis, it is most important that our polymeric magnetic nanoparticles have highly specific accumulation in pancreatic cancer cells but not in normal pancreatic cells. Therefore, the specific delivery of the polymeric magnetic nanoparticles into the pancreatic cancer cells mediated by a pancreatic cancer cell membrane receptor was investigated in vitro. Interestingly, our targeting nanoparticles presented a more specific accumulation in pancreatic cancer cells than in normal pancreatic cells, and a clear decrease in the signal intensity was observed in CKAAKN-positive BxPC-3 cells compared with CKAAKN-negative HPDE6-C7 cells and the nontargeting HA–VES@USPIO nanoparticle group. These results demonstrated that the CKAAKN–HA–VES@USPIO nanoparticles could be selectively internalized into CKAAKN-positive pancreatic cancer cells by the specific binding of the CKAAKN peptide with pancreatic cancer cell membrane receptors.

## 4. Material and Methods

### 4.1. Materials

The CKAAKN was provided from Baiaotai Biotechnology Inc. (Guangzhou, China). Trypsin was purchased from Gibco BRL, USA. Fetal bovine serum (FBS) was purchased from Sijiqing Biologic, China. The PKH67 fluorescent cell linker, 1-Ethyl-3 (3-dimethylaminopropyl) carbodiimide (EDC), N-hydroxysuccinimide (NHS), ultra-small superparamagnetic iron oxide (USPIO), 3-(4,5-dimethylthiazol-2-yl)-2,5 –diphe nyltetrazolium bromide (MTT), Coumarin 6, and cystamine were purchased from Sigma–Aldrich (St. Louis, MO, USA). Sodium hyaluronic acid (HA molecular weight: ~48,000 Da) was purchased from Freda Biochem Co., Ltd. (Shandong, China). Vitamin E succinate (VES) was purchased from Aladdin Bio-Chem Technology Co., Ltd. (Shanghai, China). All other solvents were of analytical or chromatographic grade.

### 4.2. Cell Lines

The BxPC-3 (human pancreatic carcinoma cell line) and HPDE6-C7 (normal human pancreatic duct epithelial cell line) cells were obtained from Institute of Biochemistry and Cell Biology (Shanghai, China). The BxPC-3 and HPDE6-C7 cells were maintained in Dulbecco’s Modified Eagle’s Medium (DMEM) containing 10% fetal bovine serum (Life Technologies, Inc., Carlsbad, CA, USA) at 37 °C in a humidified atmosphere containing 5% CO_2_.

### 4.3. Synthesis of CKAAKN-Functionalized Hyaluronic Acid-Based Copolymer

In the presence of EDC and NHS, the hyaluronic acid-vitamin E succinate polymer (HA–VES) was first synthesized through an amination reaction with slight modifications [28]. Briefly, 0.68 g of HA, 24.7 g of EDC, and 14.9 g of NHS were dissolved in a certain amount of PBS (pH = 5.0) under ultrasonic dispersion for 10 min. Then, the mixture was stirred at 60 °C to activate the carboxyl groups of HA. After 1 h, 1.99 g of cystamine was added into the mixture under the same conditions, and the mixture continued to react for 4 h. Then, 0.29 g of VES was dissolved in DMF and added to the reaction solution dropwise at 60 °C. The reaction occurred for 8 h, and the reaction products were transferred into a dialysis bag (7000 MWCO) against deionized water for 48 h. The final HA–VES polymer was obtained by lyophilization (SNL 315SV Speed Vac, Savant, NY, USA).

The CKAAKN was further conjugated to the surface of the HA-–VES polymer micelles. Briefly, 0.5 g of HA–VES, 0.74 g of NHS, and 1.24 g of EDC was dissolved in deionized water followed by stirring at room temperature for 12 h. Then, 20 mg of CKAAKN was added into the reaction solution with protection from light and further reacted with the activated HA–VES polymer for 10 h on a magnetic stirrer at room temperature. Finally, the reaction solution was dialyzed using a membrane (MWCO 7 kDa, Spectrum Laboratories) against deionized water for 48 h. The final CKAAKN-conjugated HA–VES (CKAAKN–HA–VES) was obtained by lyophilization (SNL 315SV Speed Vac, Savant, NY, USA) and identified by 1H-NMR spectroscopy (Bruker AV500, Swiss). The morphology of CKAAKN–HA–VES micelles was examined using transmission electron microscopy (TEM; JEOL JEM-1230, Tokyo, Japan). The size distribution of CKAAKN–HA–VES was measured using a Zetasizer (3000HS, Malvern Instruments Ltd, Malvern, Worcestershire, UK). Pyrene, a hydrophobic fluorescent probe, was used to measure the critical micelle concentration (CMC) of the micelles. The ratio of the fluorescence intensities (I_1_/I_3_, I_1_ = intensity at 374 nm; I_3_ = intensity at 385 nm) showed a cross-point as the concentration approached the CMC.

### 4.4. Preparation of USPIO-Loaded CKAAKN–HA–VES Magnetic Nanoparticles

The USPIO-loaded CKAAKN–HA–VES polymeric magnetic nanoparticles (CKAAKN–HA–VES@USPIO) were prepared using the solvent diffusion method [29]. Briefly, 50 mg of CKAAKN–HA–VES was dissolved in 10 mL of deionized water, and then 100 μL of USPIO was slowly added (5 nm, 5 mg/mL) into the solution on a magnetic stirrer, followed by stirring for 0.5 h. Then, the mixture was transferred to a round-bottomed flask to remove the volatile solvent with a rotary evaporation apparatus. The final CKAAKN–HA–VES@USPIO polymeric magnetic nanoparticles were obtained by lyophilization. The size distribution of CKAAKN–HA–VES@USPIO nanoparticles was measured using a Zetasizer (3000HS, Malvern Instruments Ltd. Malvern, Worcestershire, UK). The morphology of CKAAKN–HA–VES@USPIO polymeric magnetic nanoparticles was examined using transmission electron microscopy (TEM; JEOL JEM-1230 microscopes at 120 kV, JEM-1230, Japan). For comparison, USPIO-loaded HA–VES polymeric magnetic nanoparticles (HA–VES@USPIO, non-targeting magnetic nanoparticles) were also prepared using a method similar to that described above.

### 4.5. In Vitro Cellular Competitive Uptake Studies

Coumarin six-labeled HA–VES@USPIO and CKAAKN–HA–VES@USPIO polymeric magnetic nanoparticles were first prepared using the solvent diffusion method described above. Briefly, 50 mg of CKAAKN–HA–VES or HA–VES was dissolved in 10 mL of deionized water, then 100 μL of USPIO (5 nm, 5 mg/mL) and 50 μL of Coumarin 6 (10 mg/mL, dissolved in chloroform) were slowly added into the solution on a magnetic stirrer, followed by stirring for 0.5 h. Then, the mixture was transferred to a round-bottomed flask to remove the volatile solvent with a rotary evaporation apparatus. The final CKAAKN–HA–VES@USPIO or HA–VES@USPIO polymeric magnetic nanoparticles labeled with Coumarin 6 was obtained by lyophilization.

Before incubation with HA–VES@USPIO and CKAAKN–HA–VES@USPIO polymeric magnetic nanoparticles, BxPC-3 cells were separately stained with the PKH67 fluorescent cell linker, which can be incorporated into the cell membrane without affecting the biological activity. Briefly, the digested BxPC-3 cells were resuspended in 500 μL of diluent C, and then 500 μL of PHK67 dye (2 μM) was added. The cells were incubated for 10 min at room temperature. Then, 1 mL of serum was added to BxPC-3 cells and incubated for 2 min to stop the staining reaction, followed by centrifugation at 400× *g* for 10 min. The cell pellet was washed twice with 6 mL of complete medium to ensure the removal of unbound dye and resuspended to the desired concentration. The PKH67-labeled BxPC-3 cells were further cocultured with HPDE6-C7 cells in the same well of a 24-well plate. Then, cocultured BxPC-3/HPDE6-C7 cells were incubated with Coumarin 6-labeled CKAAKN–HA–VES@USPIO and HA–VES@USPIO nanoparticles in growth medium for 1 h. As a control, a CKAAKN-blocking experiment was carried out, in which the cocultured BxPC-3/HPDE6-C7 cells were incubated firstly with free CKAAKN (1.0 mmol/L) for 0.5 h and then with Coumarin 6-labeled CKAAKN–HA–VES@USPIO nanoparticles. The final concentration of the polymers was 50 μg/mL. After washing the cells with PBS three times, the BxPC-3/HPDE6-C7 cells were observed by confocal laser scanning microscopy (Carl Zeiss LSM 510, Jena, Germany). The intensity of the cellular fluorescence was further measured by ImageJ software.

### 4.6. In Vitro Cytotoxicity Studies

To assess the biosafety of the magnetic nanoparticles, BxPC-3 and HPDE6-C7 cells were exposed to CKAAKN–HA–VES@USPIO for 48 h, respectively. As a control, the biosafety of blank CKAAKN–HA–VES polymer was investigated in this study. According to the manufacturer’s suggested procedures (Sigma–Aldrich, St. Louis, MO, USA), cytotoxicity was measured using an MTT assay. The data are expressed as the percentage of live cells and are reported as the means of triplicate measurements.

### 4.7. In Vitro MR Imaging of CKAAKN–HA–VES@USPIO

The HPDE6-C7 and BxPC-3 cells were incubated with HA–VES@USPIO and CKAAKN–HA–VES@USPIO micelles for 1 h, respectively. After the medium was discarded, the cells were washed twice with PBS and then collected in 1.5 mL microcentrifuge tubes by centrifugation. The cells were mixed with agarose gel (0.5%, 200 μL), and T2-weighted MR images were obtained using a 3.0 T GE Discovery clinical MR750 scanner (General El ectric, Boston, MA, USA). The parameters were as followed: TR = 2000 ms, TE = 12 ms, field of view = 180 × 180 mm, slice thickness = 3.0 mm, number of slices = 8.

### 4.8. Statistical Analysis

The statistical significance of the differences between groups were assessed using the Student’s *t*-test at the significance level of *p* < 0.05 for each paired experiment.

## 5. Conclusions

In this study, we prepared a pancreatic cancer cell membrane receptor-targeting polymeric MRI nanoplatform containing USPIO for pancreatic cancer diagnosis. This work demonstrated the feasibility of using a targeting peptide to increase tumor targeting. By modifying the nanoparticles with the CKAAKN peptide, CKAAKN–HA–VES@USPIO nanoparticles could specifically internalize into CKAAKN-positive BxPC-3 cells. Interestingly, our targeting nanoparticles showed a more specific accumulation in pancreatic cancer cells than in normal pancreatic cells, and an obvious decrease in the signal intensity was observed in CKAAKN-positive BxPC-3 cells compared with CKAAKN-negative HPDE6-C7 cells and the non-targeting HA–VES@USPIO nanoparticle group. Surprisingly, CKAAKN–HA–VES@USPIO nanoparticles also exhibited excellent biosafety, and the viability of both BxPC-3 and HPDE6-C7 cells was over 80% for all concentrations of CKAAKN–HA–VES@USPIO nanoparticles after 48 h of incubation. The results demonstrated that the CKAAKN–HA–VES@USPIO nanoparticles could be selectively internalized into CKAAKN-positive pancreatic cancer cells by the specific binding of the CKAAKN peptide with pancreatic cancer cell membrane receptors. Our study provides a novel polymeric MRI contrast agent with high specificity and low toxicity for pancreatic cancer diagnosis, which makes it a very promising candidate for MRI contrast enhancement.

## Figures and Tables

**Table 1:** Particle diameter, PDI and Zeta potential of HA–VES, CKAAKN–HA–VES, HA–VES@USPIO, and CKAAKN–HA–VES@USPIO. Data represent the mean ± standard deviation (*n* = 3).

Sample	Diameter (nm)	PDI (-)	Zeta Potential (mV)
HA–VES	85.9 ± 2.6	0.385	−24.2 ± 2.2
CKAAKN–HA–VES	87.5 ± 3.5	0.415	−21.7 ± 1.9
HA–VES@USPIO	95.9 ± 5.1	0.393	−37.8 ± 3.2
CKAAKN–HA–VES@USPIO	98.2 ± 4.2	0.338	−35.4 ± 2.2

## Abbreviations

CT	computed tomography
MRI	magnetic resonance imaging
EUS	endoscopic ultrasound
PET	positron emission computed tomography
HA	hyaluronic acid
VES	vitamin E succinate
PDI	polydispersity index
FBS	fetal bovine serum
EDC	1-Ethyl-3 (3-dimethylaminopropyl) carbodiimide
USPIO	ultra-small superparamagnetic iron oxide
MTT	3-(4,5-dimethylthiazol-2-yl)-2,5–diphe nyltetrazolium bromide
BXPC-3	human pancreatic carcinoma cells
HPDE6-C7	human normal pancreatic cells
DMEM	Dulbecco’s Modified Eagle’s Medium
^1^H NMR	proton nuclear magnetic resonance
TEM	transmission electron microscope
MR	magnetic resonance
CMC	critical micelle concentration
